# A Smart Agricultural System Based on PLC and a Cloud Computing Web Application Using LoRa and LoRaWan

**DOI:** 10.3390/s23052725

**Published:** 2023-03-02

**Authors:** Mohamed Saban, Mostapha Bekkour, Ibtisam Amdaouch, Jaouad El Gueri, Badiaa Ait Ahmed, Mohamed Zied Chaari, Juan Ruiz-Alzola, Alfredo Rosado-Muñoz, Otman Aghzout

**Affiliations:** 1 Department of Computer Science Engineering, SIGL-Lab, ENSA, University Abdelmalek Essaadi, Tetouan 93153, Morocco; 2 Department of Electronic Engineering, ETSE, University Valencia, Av. Universitat, 46100 Burjassot, Spain; 3 Qatar Scientific Club, Fabrication Lab, Doha 9769, Qatar; 4 Department of Señales y Comunicaciones, University of Las Palmas de Gran Canaria, 35001 Las Palmas, Spain

**Keywords:** Industry 4.0, cloud server, IoT, smart farming, LoRa, LoRaWan, wireless monitoring, NodeRed

## Abstract

The increasing challenges of agricultural processes and the growing demand for food globally are driving the industrial agriculture sector to adopt the concept of ‘smart farming’. Smart farming systems, with their real-time management and high level of automation, can greatly improve productivity, food safety, and efficiency in the agri-food supply chain. This paper presents a customized smart farming system that uses a low-cost, low-power, and wide-range wireless sensor network based on Internet of Things (IoT) and Long Range (LoRa) technologies. In this system, LoRa connectivity is integrated with existing Programmable Logic Controllers (PLCs), which are commonly used in industry and farming to control multiple processes, devices, and machinery through the Simatic IOT2040. The system also includes a newly developed web-based monitoring application hosted on a cloud server, which processes data collected from the farm environment and allows for remote visualization and control of all connected devices. A Telegram bot is included for automated communication with users through this mobile messaging app. The proposed network structure has been tested, and the path loss in the wireless LoRa is evaluated.

## 1. Introduction

The fourth industrial revolution, commonly known as “Industry 4.0”, has emerged as a hot topic of research and discussion among industry and academia, especially in the fields of management and engineering [1,2]. Industry 4.0 refers to a new industrial paradigm that encompasses various technologies like Artificial Intelligence (AI), Augmented Reality (AR), big data, remote sensing, and the Internet of Things (IoT) that play a crucial role in increasing productivity and reducing costs across different industries including the agricultural sector [3,4,5]. The integration of Industry 4.0 with agriculture has led to the emergence of smart agricultural and farming systems.

In agriculture, relying on traditional farming methods to meet the growing demand for food is no longer sufficient. The production process of planting, sowing, reaping, irrigation and cultivation must now respect certain climatic conditions, such as air temperature, humidity, and precipitation. These conditions affect the spread of pests and diseases, which cause significant losses in global food production [6]. In fact, around 40% of global food production is lost due to pests and diseases infecting the plants [3]. In addition, over-irrigation and leakages in water channels result in the wastage of around 20% of water reserved for agricultural activities due to many reasons such as leakages and line-losses in the waterway channels and the over-irrigation [7].

To address these issues, smart agricultural and farming systems that integrate IoT and communication models are being introduced. These systems automate farm operations in a collaborative and intelligent manner, improving the reliability of crop production management. They provide better control of planting conditions and natural resource utilization without human interaction. The smart farm environment enables decision making by gathering information from different sensors and analyzing it according to needs. The automation of certain processes using IoT sensors, communicating devices, control units, and computers replaces manual work schedules and improves production. As smart farming systems help the interoperability of multiple heterogeneous devices, information sharing and processing is important for controlling the operations of the farm. Multiple sensors are deployed in the smart farm such as humidity and temperature sensors forming a Wireless Sensor Network (WSN). These sensors collect the environment information and send it to the server where data are processed. To deploy a large-scale WSN, Low-Power Wide-Area Network (LPWAN) technologies such as LTE, SigFox and LoRa are better suited due to their wide transmission range, scalability and low power consumption [8,9]. LoRa technology is among the most commonly used technologies in smart farming, owing to its long range and low power consumption, as well as its use of a free-licence band (863 to 870 MHz in Europe) [10] which allows transmitting data over several kilometres in rural areas without expensive infrastructure or cellular connectivity. LoRa devices also have low power requirements, meaning they can operate on battery power for years, making them ideal for remote and hard-to-reach areas of a farm. Additionally, LoRa devices are relatively inexpensive, making them a cost-effective solution for smart farming applications. They are easy to install and configure, and can be integrated with a wide range of sensors and devices.

This paper presents a novel approach to integrating LoRaWAN communication with traditional Programmable Logic Controllers (PLCs), which have long been used in agriculture for automating various processes and controlling machinery. The proposed system facilitates the creation of smart farms by enabling the integration of LoRa connectivity with the existing automated processes that have been in use for decades, without the need for replacing the old control systems. To accomplish this integration, the proposed system employs a LoRa shield on a Simatic IOT2040, which communicates with the existing PLC using the Modbus-TCP protocol. The resulting system allows us to control the operation of farming machines like water pumps and collects data from different sensor nodes located throughout the farm. A cloud server is utilized for data processing, offering a secure, flexible, and scalable web-based platform that provides a user-friendly interface for remotely managing all the devices in the smart farm system.

This innovative approach has the potential to significantly benefit the agriculture industry. By incorporating LoRa connectivity into the existing PLCs, the system can leverage previously automated processes and thus reduce the need for expensive replacement of old control systems. The remote management capabilities can help reduce labor costs and increase productivity. In addition, the real-time monitoring of climatic conditions, such as temperature and humidity, can assist in detecting and preventing the spread of pests and diseases, thereby contributing to the overall improvement of global food production.

## 2. Literature Review

Certainly, recent studies have highlighted the growing interest in using LoRa technology for smart farming applications. Placidi et al. demonstrated in [11] the use of a LoRa-based soil moisture monitoring system for precision agriculture in smart cities. Another study of Boursianis et al. published in [12] investigated the development of a smart irrigation system for precision agriculture based on LoRaWAN technology. Another study presented by Behjati et al. in [13] explored the use of drones towards large-scale livestock monitoring in rural farms. Widianto et al. in [14] presented a Systematic Review of Current Trends in Artificial Intelligence for Smart Farming to Enhance Crop Yield. Jiang et al. [15] proposed a fully customized low-cost and low power smart farming network structure enabled by LoRa and ANT radios. Yoon et al. [16] proposed a smart farm based on LoRa & MQTT. In [17], Escolar et al. proposed a LoRa-based network of energy-harvesting devices for smart farming. Kodali et al. [18] described a smart irrigation system based on LoRa technology. Furthermore Ramli et al. [19] presented an adaptive network mechanism for a smart farm system by using LoRaWAN and IEEE 802.11ac protocols. These studies demonstrate the growing interest in using LoRa technology for smart farming applications and the potential benefits that it can offer.

## 3. Material, Methods and Experimental Tests

A smart farm can be defined as a new type of automated farming system using IoT infrastructure. Our proposed system consists of four main parts: the end-node sensors, an IoT LoRaWan gateway, the control equipment, a cloud server hosting a web-based platform for control and monitoring, and a bot for a Telegram messaging application in mobile devices. In this section, we present a brief overview of LoRa and LoRaWan, and then we describe the proposed smart farm system.

### 3.1. LoRa and LoRaWan Overview

LoRa and LoRaWAN are global de facto standards of Low-Power Wide Area Networks (LPWANs), with LoRa being the physical layer and LoRaWAN the Media Access Control (MAC) layer. LoRaWAN is an open specification developed by the LoRa Alliance [20]. LoRa technology is based on Chirp Spread Spectrum (CSS) modulation, which offers high sensitivity for the receiver and robustness against data corruption through the use of forward error correction messages [21]. LoRa uses unlicensed radio spectrum in the Industrial, Scientific, and Medical (ISM) band, specifically the 863–870 MHz range in Europe. The communication range and robustness of LoRa signals are affected by various parameters such as transmission power, Spreading Factor (SF), which is the ratio between the data symbol rate and chirp rate, and Code Rate (CR), which is the forward error correction rate that affects packet transmission airtime and bandwidth [22]. LoRa modulation uses six orthogonal Spreading Factors, ranging from 7 to 12, with a trade-off between a higher data rate and a longer range or lower power consumption. Lower SF results in faster chirps, higher data transmission rates, shorter active times for the radio transceivers, and longer battery life. However, like any technology, LoRa has its disadvantages. One of the main drawbacks is its limited bandwidth, which can result in slow data transfer rates. LoRa’s data rate is also fixed, which can be a problem when trying to transmit large amounts of data. Additionally, LoRa suffers from interference issues, as it operates in an unlicensed spectrum that is shared with other wireless technologies. This can cause communication problems, particularly in urban environments with high levels of radio frequency activity. Table 1 summarizes the pros and cons of the LoRa technology:

The network of LoRa uses encryption, integrity and authentication and it is secured with two security layers [23]:

- Network layer: an AES-128 secret key named network session key (NwkSKey) is shared between the end-device and the network server for authentication.
- Application layer: an AES-128 secret key named application session key (AppSKey) protects the payload transmission between end-devices and the application server.

In general, the architecture of a LoRa-based network consists of a hierarchical topology, formed by LoRa nodes, gateways network servers, and application servers. The devices can transmit the data to the gateways which belong to the same LoRaWan network. However, all gateways within the range of a LoRa node can receive messages and the duplications (when existing) will be filtrated in the network server responsible of processing the incoming packets.

### 3.2. Smart Farm Infrastructure

**Figure 1:**
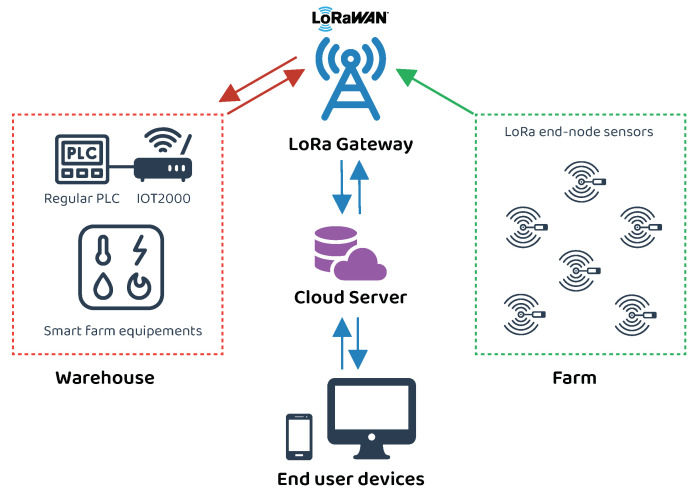
Smart farm system architecture overview. The farm is connected to a LoRa gateway which exchanges data with users via a cloud server.

The proposed smart farm system consists of two main networks as described in Figure 1:

1. The monitoring network (Farm): a wireless network of different LoRa sensors distributed over the farm to collect to the required information such as moisture and airflow sensors.
2. The control network (Warehouse): The reaped vegetables and fruits are stored in the warehouse. Controlling the climatic conditions of this place is a must, thus it is equipped with different environmental sensors (i.e., temperature sensor). The warehouse contains all the control equipment such as air conditioner and irrigation water pump. These devices are controlled with a PLC.

The LoRa sensors scattered in the farm send the sensed information (pH, moisture, air flow, temperature, etc.) to the LoRa gateway. The gateway is connected to the internet and it is responsible for forwarding the sensor data to the cloud server to be further analyzed and stored in the database. The gateway is also responsible of receiving the control commands from the server and forward them to the PLC located in the warehouse. These commands can be manually instructed by the user through the web-based platform or automatically executed if the sensed received data require an action (e.g., the PLC will activate the water pump if the soil needs more water). The automatic commands can be set and configured by the user in the web platform by defining the target device and the threshold values that trigger the actuating device. For better communication coverage, the LoRa gateway should be placed in a high altitude and all the LoRa end-nodes have to be distributed properly.

**Figure 2:**
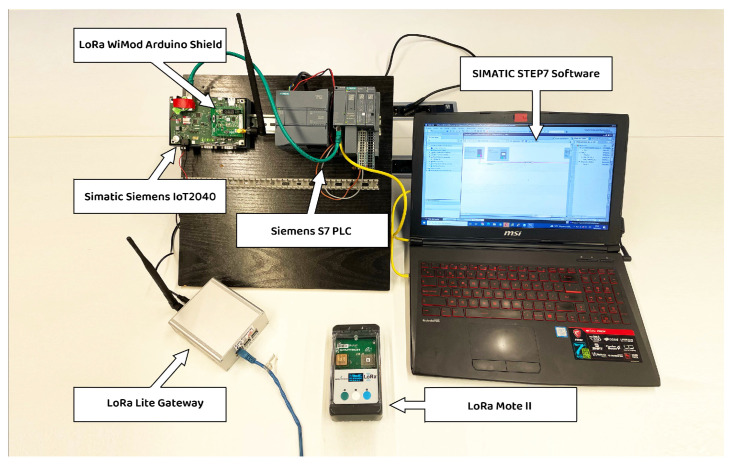
Siemens S7 PLC connected to Siemens Simatic IOT2040 incorporating the Wimod LoRa shield. The LoRa gateway is powered and connected to the internet. The inputs/outputs of the different devices in the farm are simulated with the Simatic Step7 software and controlled with the S7 Siemens PLC.

In IoT, a gateway acts as a bridge between devices and the cloud or server. The gateway is responsible for collecting data from sensors and devices, processing it, and sending it to the cloud or server for storage and analysis. Here are some of the gateway protocols used in IoT such as MQTT, CoAP, HTTP and DDS [24]. In the case of LoRaWan technology, the gateway uses the LoRaWAN protocol, which is responsible for managing the communication between the end devices and the LoRa gateway, as well as providing security, data encryption, and authentication. It also provides the network architecture that enables communication over long distances with minimal power consumption, making it ideal for IoT applications. In order to establish the LoRa communication in our system, we used the ”WiMOD LoRa Lite Gateway“ from IMST. The warehouse contains the PLC to control the devices in the farm such as the water pump. This PLC should be wirelessly connected via LoRa so it can establish a connection with the gateway to receive the commands from the cloud server. To achieve this, we have connected the Simatic IOT2040 from Siemens [25] to a regular S7 Siemens PLC via Modbus TCP. The LoRa Wimod Arduino shield board from IMST is inserted in the Simatic IOT2040 as additional communication board, serving as LoRa end-node in the same way as other LoRa end-node sensors in the farm (e.g., Mote II board or custom-made LoRa end-nodes). This solution makes it possible to connect existing PLCs to the LoRa network. The devices used in our test bench are illustrated in Figure 2. The inputs/outputs of the devices of the farm are simulated with the SIMATIC Step7 software and controlled with the S7 Siemens PLC.

**Figure 3:**
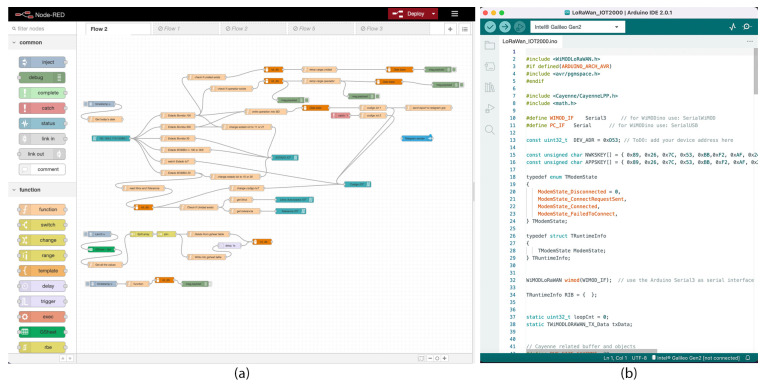
(**a**) NodeRed dashboard, it provides a browser-based editor that makes it easy to wire together flows using the nodes. (**b**) a part of the code used to program the LoRaWan communication of the SIMATIC IoT2040.

The Siemens Simatic IOT2040 operates with Yocto Linux and it can be easily expanded with Arduino shields in a compact industrial design. The applications can be easily programmed using Node-Red visual programming tool as described in Figure 3a. The WiMOD Shield is a expansion board that enables users of Arduino-compatible boards to use WiMOD radio modules based on LoRa. The Shield includes everything that is needed for connecting a WiMOD module to an Arduino board by using the WiMODLoRAWAN library. The shield offers two UART connections, able to communicate with the IOT2040 main board. The LoRa communication shield can be programmed using the Arduino IDE software as described in Figure 3b, and then writing the code directly to the IOT2040 using a USB cable thanks to the Intel Galileo firmware which should be added to the Arduino IDE first. In the NodeRed dashboard, we have added the “*node-red-node-arduino*” package in order to read the LoRa payloads exchanged between the IOT2040 and the gateway through the LoRa shield board. The “*node-red-contrib-s7*” package is used to read/write commands to the Siemens S7 PLC via Modbus-TCP (any other PLC can also be connected using standard Modbus-TCP wired communication). A part of the Arduino code and the NodeRed scheme of the smart farm are represented in Figure 3.

The PLC in the warehouse controls the following elements:

- Water pump and associated valves: controls the amount of water needed for irrigation.
- Air conditioner and fans for ventilation and air quality, maintaining an adequate level of temperature and humidity to preserve food products.
- Intrusion alarms.
- Lighting: on/off programming for presence simulation and night activity.
- LoRa Temperature sensor.
- LoRa Humidity sensor.

The Siemens S7 PLC is controlled using the IOT2040 via Modbus-TCP, which also exchanges commands and data with the LoRa gateway. The rest of sensors installed in the farm act as LoRa end-nodes and directly communicate with the LoRa gateway.

In this system, a bot was used to integrate Telegram instant messaging using Telegram Bot API in both the IOT2040 and the web application. The Telegram bot was made by registering to @botfather (https://telegram.me/BotFather accessed on 2 February 2023). There are steps that must be completed in @botfather, such as creating a bot name, bot username, and command with command */newbot*. A bot token was used to communicate with our system via Telegram once the bot was created.

The first integration of the Telegram bot with the IOT2040 was done by installing the NodeRed package “*node-red-contrib-telegrambot*”. This integration aims to receive direct control commands from users, for example, turning on the lights. The Telegram node was programmed with a JSON code to define several commands and their actions. Thus, the user can send his command via telegram, the IOT2040 will analyse the request, make it, and send a confirmation message to the user.

The second integration of the Telegram bot is done with the web application described in Section 5 using the *Telegram Bot API—PHP SDK* [26]. This integration is done using the same token as the IOT2040 in order to use the same telegram bot for both integrations. The aim of this second integration is to analyse the user requests related to the database (e.g., request the last irrigation water usage, request the time that the lights were switched off, etc.), or the request related to the sensor nodes in order to receive the instant measured value of a sensor without waiting the duty cycle time needed to send the last measured value to the database.

In this work, The Things Network (TTN) is used for LoRaWan communication. The LoRa end-nodes and gateways based on LoRaWan standards can exchange data using the TTN for free since it is an open source infrastructure [27]. Nowadays, many users around the world have registered thousands of gateways in the TTN platform to broaden the network in a collaborative way. The users can connect their IoT LoRa devices to the already existing gateways in the TTN network. Also, establishing a connection with a private server is also possible if a user wants to use his own specific platform.

### 3.3. Experimental Test

The experimental test was conducted throughout a farmland in Valencia city, Spain. In this test, we used a LoRa Mote II from IMST as end-node, which incorporates an accelerometer, an altimeter, a temperature sensor and a GPS module. The LoRa gateway was located in the balcony of an apartment, in the 6th floor, with an altitude of 24 m, approximately.

The configuration used for the LoRa end-node device was:

- LoRa Class: Class A
- Frequency: 867.1–868.5 MHz
- Bandwidth size: 125 kHz
- Spreading Factor (SF): 7
- Coding Rate: fixed = 4/5
- Transmitting Power: +14 dB
- Transmitting Antenna gain: +2 dB
- Receiving Antenna gain: +2 dB

**Figure 4:**
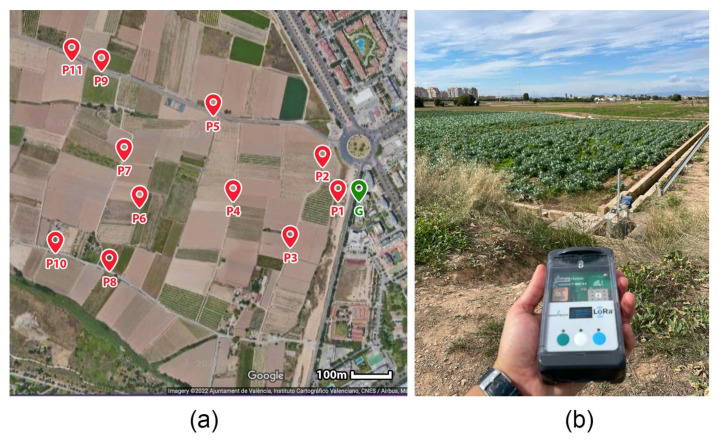
Experimental setup for outdoor LoRa communication test. (**a**) P1 to P14 represent the measurement locations, G represent the gateway location. (**b**) LoRa Mote II used as LoRa end-node.

The LoRa end-node was placed in several locations with different distances from the gateway. From each transmit location, 10 messages were exchanged between the end-node and the gateway. The location of the LoRa gateway and the different measurement positions (P1 to P11) are represented in Figure 4. The Spreading Factor (SF) was fixed in 7 in order to reach the maximum distance. However, in similar applications it is preferred not to fix the SF and set Adaptive Data Rate (ADR) mechanism for optimizing data rates, airtime and power consumption, and consequently improve the range and capacity of the network.

The Received Packets Percentage (RPP) was calculated in each location as described in Equation (1), where NACK denotes the number of packets with ‘Acknowledgement’ signal received, and NAP denotes the number of all transmitted packets.

(1) $$RPP\left[\%\right]=100\times \frac{NACK}{NAP}$$

Studying Path Loss (PL) is important because it helps to understand how the strength of a LoRa signal decreases as it travels through the environment. This information is useful for predicting the range of a LoRa network and for designing efficient communication systems. By understanding the factors that contribute to LoRa path loss, such as the type of environment, the distance between the transmitter and receiver, and the frequency of the signal. Modeling the PL predict the reduction in power of a signal as it propagates through a medium, and it can help for better distribution of the end-nodes and identify potential issues with a LoRa network and suggest ways to improve its performance.

The Large-Scale Fading (LSF) is characterized by its Path Loss parameter (PL). The PL has been evaluated in outdoor environments on the basis of the measured RSSI and signal-to-noise ratio (SNR) using Equation (2) [28], where $P_{t}$ is the transmission power $G_{t}$ and $G_{r}$ are the gains of the transmitting and receiving antennas, respectively.

(2) $$PL=P_{t}+G_{t}+G_{r}+10\times \log_{10}\left(1+\frac{1}{SNR}\right)-RSSI$$

The ratio between the received power $P_{r}$ and the transmitted power $P_{t}$ in a free space environment is given by the Friis law (Equation (3)), where $G_{t}$ and $G_{r}$ are the gains of the transmitter and the receiver, respectively; λ is the wave length, and *d* is the distance between the receiver and the transmitter.

(3) $$\frac{P_{r}}{P_{t}}=G_{t}G_{r}{\left(\frac{\lambda }{4\pi d}\right)}^{2}$$

For a non-free space environment, a path loss exponent γ and a reference distance are introduced. Then, Equation (3) becomes Equation (4).

(4) $$\frac{P_{r}}{P_{t}}=G_{t}G_{r}{\left(\frac{\lambda }{4\pi }\right)}^{2}\times \frac{1}{d_{0}^{\gamma }}\times {\left(\frac{d_{0}}{d}\right)}^{\gamma }$$

Using the first-order fit [29], PL can be estimated by modelling the experimental PL. Then, the Estimated Path Loss (EPL) can be obtained by Equation (5).

(5) $$EPL=PL_{0}+10\times \gamma \times log_{10}\left(\frac{d}{d_{0}}\right)$$

The $PL_{0}$ in microcellular systems is the PL intercept at a reference distance $d_{0}$, typically ranging from 1 m to 100 m. In this paper, we consider $d_{0}$ = 1 m. The path loss exponent γ can be estimated by analyzing the measurement results of the propagation environment. This can be done by fitting a model to the measured data and extracting the relevant parameters, including the path loss exponent. The specific method used may vary based on the type of propagation environment and the information available [30]. Typically, in free-space, gamma is 2. In urban environments, it ranges from 2.7 to 3.5, while in building environments, it can vary from 1.6 to 6 based on building structure, materials, and obstacles [23]. There may be a difference between the actual path loss (PL) and estimated path loss (EPL) due to shadow fading deviation, as shown in Equation (6). When presenting PL data, there are several statistical measures that can be used to provide a more complete picture of the data. Some of the most commonly used statistical measures include Mean, Standard deviation and Range. The statistical measures and the EPL parameters are represented in Table 2.

(6) $$\sigma_{SF}=std\left(PL-EPL\right)$$

## 4. Results and Discussion

In the previous test, measurement points with RPP below 50% are ignored. The Received Signal Strength Indicator (RSSI), the signal-to-noise ratio (SNR) and the percentage of the received packets (RPP) corresponding to each measurement point are represented in Table 3.

**Figure 5:**
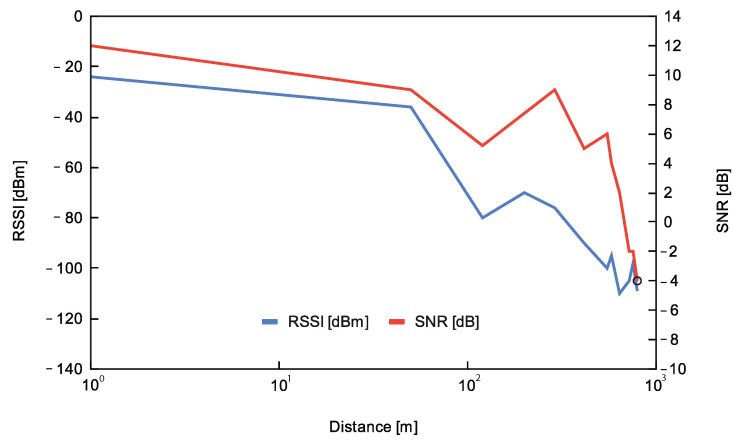
LoRa communication values in outdoor testing: Average RSSI [dBm] and Average SNR [dB] as a function of distance [m].

The RSSI and SNR variation as a function of the distance are illustrated in Figure 5. As seen, the RSSI decreases as the distance increases. The first packet loss was at position P6 (550 m); from position P5 and above, the RSSI decreased under −90 dBm. Concerning SNR, it followed a similar trend: the SNR value stayed above 0 dB until P9 (720 m) and then, it decreased to −4 dB at P11 (795 m). The lowest rate was registered at P8 (640 m), whereas at P11 (795 m) the received packets reached 60%. This test shows that distances under 500 m can guarantee successful communication between end-nodes and the gateway.

**Figure 6:**
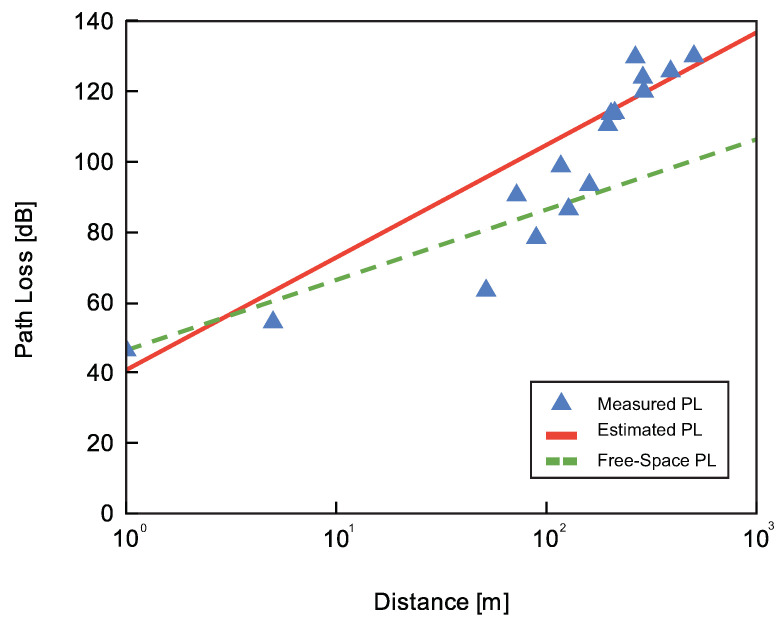
Estimated and measured PL compared with the FS model in the farm environment.

Using the experimentally collected data of Table 3, the PL was calculated using the Equation (2). The results are compared with the Estimated Path Loss (EPL) along with the Free-Space Path Loss (FSPL) model and are illustrated in Figure 6. These results shows he proposed model to estimate the path loss appears to be more valid as the distance increases. For distances shorter than 70m, the proposed model shows instability that mainly return to the shadowing effects [28]. Packets with a PL lower than 126 dB can be received. As would be expected, the free space model FSPL shows the lowest attenuation.

As a result, the PL model parameters are typically only valid in specific environments, frequency ranges, and antenna configurations. Thus, for similar condition to our experimental test, the proposed PL estimation model can be applied in the design of a LoRa mesh network in a farm to improve the energy efficiency and reliability over existing LoRa systems. LoRa is designed to operate at very low power levels, which is one of the reasons why it is able to achieve long-range communication. However, this also means that the signal is more susceptible to interference and noise as it propagates through the environment, which can cause a sudden and significant increase in PL. The LoRa signal may encounter a variety of environmental factors that can cause a sudden increase in path loss, such as obstacles (e.g., buildings, trees) and interference from other wireless devices. These factors can cause the signal to be absorbed, scattered, or reflected in different directions, resulting in a significant increase in PL.

The transmission range of LoRa in agriculture is an important factor to consider when implementing smart farming solutions. The range of LoRa depends on various factors, such as the frequency used, transmission power, terrain, and obstacles present. In general, LoRa has been found to have a transmission range of several kilometers in rural areas and up to several hundred meters in urban environments. This range is suitable for most smart farming applications, which typically cover a large area [31]. However, in some cases, the transmission range of LoRa may need to be extended to cover a larger area. This can be achieved by using multiple LoRa gateways, which act as intermediate nodes between the end devices and the central control system. LoRa gateways can receive and forward messages over long distances, thereby extending the transmission range of the end devices.

Recent studies have demonstrated the potential of LoRa for smart farming [31,32]. These studies have highlighted the use of LoRaWAN for remote monitoring of large agricultural areas [32], as well as the development of systems based on LoRa for monitoring the agricultural sector [33]. Additionally, Semtech’s LoRa technology has been used to show significant improvements in smart agriculture use cases, such as a 50% water savings [34].

In conclusion, LoRa is a promising technology for smart farming applications. It offers long-range, low-power communication that can enable the collection of data from various sensors and devices over a wide area, making it an ideal solution for precision agriculture.

## 5. Web-Based Monitoring Platform

The presented smart farm system requires a tailored application to customize the control and analysis of data and provide more flexibility in its management. To meet this need, we have developed a personalized web application called ‘Mi granja (My Farm)’, using the Laravel framework [35] for back-end operations and a MySQL database. We decided to implement a relational database due to its ability to provide easier management and maintenance of data quality and integrity over time. This is due to features such as data consistency, query flexibility, transaction support, and scalability, which can be beneficial for the long-term sustainability and robustness of the application. Alternatively, a non-relational database could also be utilized for this purpose. The front-end of the web application is developed using the Bootstrap framework, which implements a responsive design that is supported by multiple screen sizes. The web application allows users to easily access and analyze the data collected by the sensor nodes, as well as remotely control the machines connected to the PLC. It also provides a user-friendly interface for configuring the system settings and defining custom alerts and notifications. The web application is hosted on a cloud server, enabling users to access it from anywhere with an internet connection.

**Figure 7:**
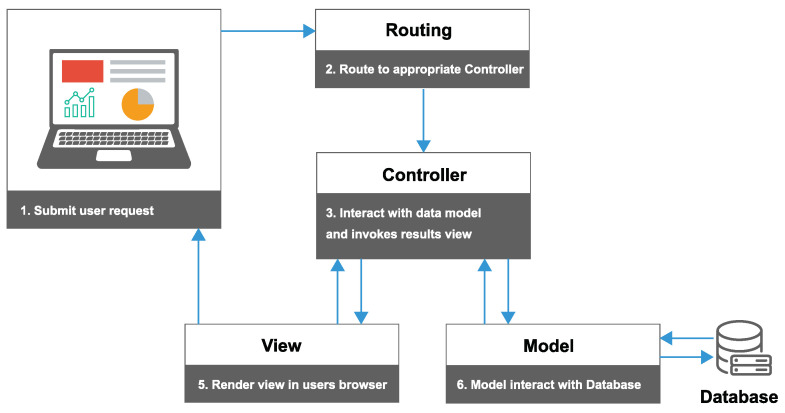
The architecture of the web monitoring platform illustrates the flow of actions from the user’s request to the point of receiving the response.

In Laravel, the Model-View-Controller (MVC) architecture is used to separate the application logic from the presentation layer. The work process of the MVC architecture. Routing is the process of accepting a request and directing it to the appropriate controller is described in Figure 7. Route model binding provides a convenient way to automatically inject the model instances directly into your routes. Controllers are responsible for handling user requests and retrieving or storing data in the database through Models which are used also to pass this data off to a view. Implicit route model binding is a smart feature in Laravel that can resolve Eloquent models defined in routes or controller actions and whose values are passed as parameters to controller methods. In this work, the database is running MySQL to store the data in the different tables and the user interface is based on Bootstrap framework and Angular Js that consist of a set of Cascading Style Sheets (CSS) classes and JavaScript functions, providing a responsive design supporting different screen sizes. The interface includes a navigation system and a graphical content displayed in the form of charts and tables to display historical data. The open-source library CanvaJs was used to create the charts. The platform also allow users to export data in different formats for further analysis by off-line software tools.

The LoRa gateway communicates with the TTN server, which will forward the received payloads directly to our cloud server to be stored in the database, so that it can be visualized and monitored from the user interface. The user commands also take the same path: they are transmitted to the TTN server and then forwarded to the LoRa gateway, which will finally communicate with the LoRa end-node. The web application allows different users to create their accounts, each user can register several networks corresponding to his associated farms (multiples farms can be managed from the same web interface), and each network (each farm, in fact) contains a number of connected devices. In order to register a device in the network, the user should register this device in the TTN server first, and then register it in the dashboard page of the web application using its identifier, because all the data go through the TTN LoRaWan network before receiving/sending it to our cloud server.

**Figure 8:**
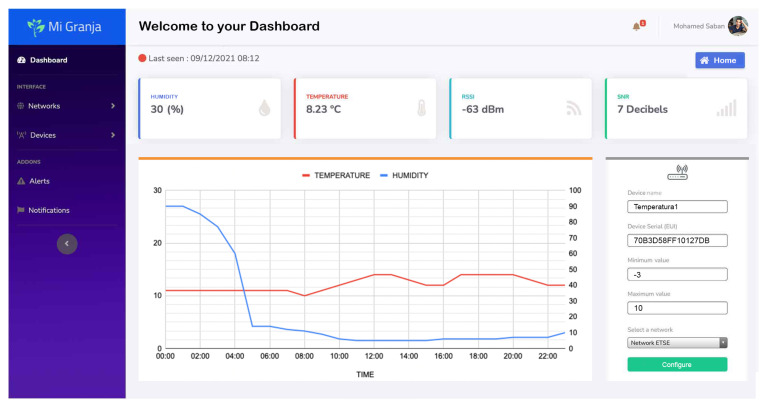
Screenshot from the web application “Mi Granja” showing the dashboard page and relevant data of a temperature sensor.

The home page and dashboard of the web application is illustrated in Figure 8. On the dashboard, the user can define multiple parameters related to each LoRa end-node, such as maximum and minimum values for specific devices. If the received value is outside the defined range, the user will receive an alert notification via email and Telegram. These notification and alert messages can be customized in the dashboard. The data collected by the sensor nodes can be viewed in the form of tables and charts and can be downloaded as an Excel or CSV file for manual and advanced analysis if needed. The web application also has the flexibility to easily add additional features thanks to its MVC design based on the Laravel framework. Overall, the web application provides a comprehensive and user-friendly interface for managing and analyzing the data collected by the smart farm system.

The web application is hosted in a cloud server and its main features are:

- Scalable design, light weight and fast response.
- Strong security thanks to the MVC design and authentication system.
- Multi-device experience: users can access to the web application from various devices.
- Instant messaging: users receive notifications via email and Telegram.
- Different charts and graphs for Data visualisation.
- Organized data storage and easy searching.
- Export data for advanced analyses as Excel and CSV files.

## 6. Conclusions

This paper outlines the development of a comprehensive IoT system for a smart farm. The system aims to improve food product storage by monitoring and regulating humidity and temperature levels. It also enables remote monitoring and control of various devices through a web-based application, automating tasks such as irrigation and temperature adjustments.

The developed web application is designed to simplify the process of creating and managing IoT networks. It provides users with the ability to add devices and access to data analysis and downloading features. The use of the MVC design pattern makes the application easy to modify and update, ensuring scalability and the ability to add new features as needed. The responsive design of the app makes it accessible on different screen sizes, ensuring a seamless user experience on any device. The low server resource consumption allows the app to support a large number of concurrent users, making it ideal for managing multiple sensor networks. The introduction of new additions to the traditional LoRa IoT network infrastructure, such as the LoRa link for exchanging data with automation PLCs commonly found in farms and the Telegram link for direct messaging with users via popular messaging apps, further enhances the functionality of the system.

**Figure 9:**
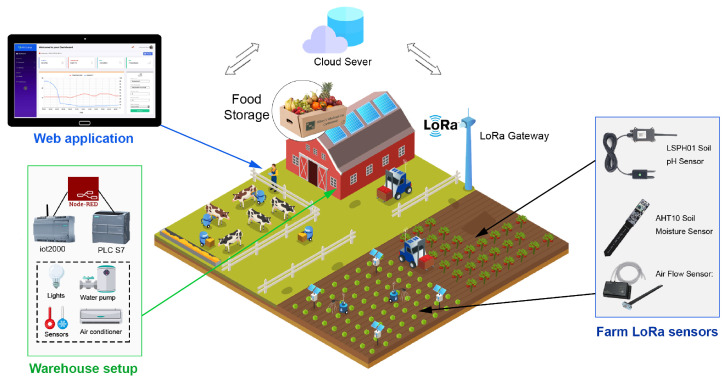
Illustration of the smart farming application. The proposed IoT system integrates different devices and technologies: PLC controllers and LoRa nodes connected via a gateway. The gateway forward and receive data from the cloud server that hosts the web application.

The general illustration of the presented system is described in Figure 9 [3]. The smart agricultural system utilizes LoRa end-node sensors scattered throughout the farm to collect data about the environment. The sensors transmit this data to a LoRaWAN gateway, which then sends it to a cloud server for analysis. The system also includes a Programmable Logic Controller (PLC) that is connected to various machines in the warehouse, such as water pumps and lights. The IoT2040 is connected to the PLC via Modbus-TCP and programmed with Node-RED. Users can access the system through a web-based monitoring application, which allows for remote control of the warehouse machines through the IoT2040. Additionally, users can send commands and requests via a Telegram bot. The cloud server analyzes the received information and stores useful data.

To conclude, the proposed infrastructure represents a smart solution for farmers to integrate the IoT in their already existed farming systems which mainly are relying on a regular PLC. Currently, we are working on the development of some Artificial Intelligence (AI) data analysis tools that can be added to the presented systems for better farm management.

## Figures and Tables

**Table 1:** The pros and cons of of implementing LoRa and LoRaWAN technologies.

Pros	Cons
Long range	Low data transfer rate
Low power consumption	Limited capacity
Low cost	Interference (unlicensed frequency band)
Security	Centralized architecture
Scalability and flexibility	High latency

**Table 2:** Lognormal PL Model parameters in the outdoor environment and statistical measures.

EPL exponent (γ)	2.9
EPL intercept (PL($d_{0}$))	36.34 dB
Standard deviation ($\sigma_{SF}$)	6
Standard error of the mean (SEM)	1.81
Range	71.29 dB

**Table 3:** SNR, RSSI and RPP of received packets from each measurement point.

Locations	Distance (m)	RSSI (dBm)	SNR (dB)	RPP (%)
P1	50	−36	9	100
P2	120	−80	5.2	100
P3	200	−70	7.4	100
P4	290	−76	9	100
P5	415	−90	5	100
P6	550	−100	6	90
P7	580	−95	4	80
P8	640	−110	2	50
P9	720	−105	−2	60
P10	755	−98	−2	70
P11	795	−109	−4	60

## Data Availability

Not applicable.

## Abbreviations

The following abbreviations are used in this manuscript:

PLC	Programmable Logic Controllers
IoT	Internet of Things
AR	Augmented Reality
AI	Artificial Intelligence
WSN	Wireless Sensor Network
LPWAN	Low-power wide-area network
CR	Code Rate
SF	Spreading Factor
CSS	Chirp Spread Spectrum
NwkSKey	Network session key
AppSKey	Application session key
UART	Universal asynchronous receiver-transmitter
TTN	The Things Network
ADR	Adaptive Data Rate
RPP	Received Packets Percentage
RSSI	Received Signal Strength Indicator
SNR	Signal-to-noise ratio
PL	Path Loss
EPL	Estimated Path Loss
FSPL	Free-space Path Loss
LSF	Large-Scale Fading
MVC	Model-View-Controller
